# Supplementary material for: L-shaped relationship between dietary niacin intake and hearing loss in United States adults: National health and nutrition examination survey
Source: PLoS One. 2025 Feb 25;20(2):e0319386. doi: 10.1371/journal.pone.0319386 (PMC11856504; doi:10.1371/journal.pone.0319386)
Supplement: S1 Table — (DOC) [file pone.0319386.s001.doc]

**S1 Table Baseline characteristics of included and excluded participants**

| **Variables** | **Total (n = 9444)** | [**Exclude**](javascript:;)**d(n = 1769)** | [**Included**](javascript:;)**(n = 7675)** | **p** |
| --- | --- | --- | --- | --- |
| **Sex,n (%)** |  |  |  | < 0.001 |
| **male** | 4582 (48.5) | 773 (43.7) | 3809 (49.6) |  |
| **female** | 4862 (51.5) | 996 (56.3) | 3866 (50.4) |  |
| **Age, Mean ± SD,years** | 44.0 ± 14.3 | 43.8 ± 14.3 | 44.0 ± 14.3 | 0.685 |
| **Race/ethnicity,n (%)** |  |  |  | < 0.001 |
| **Non-Hispanic white** | 2918 (30.9) | 382 (21.6) | 2536 (33.0) |  |
| **Non-Hispanic black** | 2320 (24.6) | 413 (23.3) | 1907 (24.8) |  |
| **Mexican American** | 1372 (14.5) | 249 (14.1) | 1123 (14.6) |  |
| **Others** | 2834 (30.0) | 725 (41.0) | 2109 (27.5) |  |
| **Education level ,n (%),years** |  |  |  | < 0.001 |
| **<9** | 862 ( 9.1) | 257 (14.5) | 605 (7.9) |  |
| **9-12** | 1209 (12.8) | 261 (14.8) | 948 (12.4) |  |
| **>12** | 7373 (78.1) | 1251 (70.7) | 6122 (79.8) |  |
| **Marital status,n (%)** |  |  |  | 0.383 |
| **Married or living with apartner** | 5637 (59.7) | 1071 (60.6) | 4566 (59.5) |  |
| **Living alone** | 3801 (40.3) | 695 (39.4) | 3106 (40.5) |  |
| **Family income, n (%)** |  |  |  | < 0.001 |
| **Low** | 2941 (34.3) | 626 (41.9) | 2315 (32.7) |  |
| **Medium** | 3067 (35.7) | 470 (31.4) | 2597 (36.6) |  |
| **High** | 2576 (30.0) | 399 (26.7) | 2177 (30.7) |  |
| **BMI ,Mean ± SD,kg/m^2^** | 29.3 ± 7.2 | 28.3 ± 7.0 | 29.5 ± 7.2 | < 0.001 |
| **Smoking status,n (%)** |  |  |  | < 0.001 |
| **Never** | 5580 (59.1) | 1115 (63.1) | 4465 (58.2) |  |
| **Current** | 2031 (21.5) | 369 (20.9) | 1662 (21.7) |  |
| **Former** | 1823 (19.3) | 283 (16.0) | 1540 (20.1) |  |
| **drink status,n (%)** |  |  |  | < 0.001 |
| **Yes** | 5813 (72.3) | 459 (62.7) | 5354 (73.2) |  |
| **No** | 2229 (27.7) | 273 (37.3) | 1956 (26.8) |  |
| **Hearing protection,n(%)** |  |  |  | < 0.001 |
| **Always** | 670 ( 7.1) | 105 (5.9) | 565 (7.4) |  |
| **About half the time** | 625 ( 6.6) | 91 (5.2) | 534 (7.0) |  |
| **Seldom** | 519 ( 5.5) | 67 (3.8) | 452 (5.9) |  |
| **Never** | 7625 (80.8) | 1503 (85.1) | 6122 (79.8) |  |
| **Diabetes,n(%)** |  |  |  | 0.080 |
| **Yes** | 1069 (11.3) | 179 (10.1) | 890 (11.6) |  |
| **No** | 8369 (88.7) | 1587 (89.9) | 6782 (88.4) |  |
| **Hypertension, n (%)** |  |  |  | 0.012 |
| **Yes** | 2883 (30.6) | 496 (28.1) | 2387 (31.1) |  |
| **No** | 6553 (69.4) | 1271 (71.9) | 5282 (68.9) |  |
| **Coronary heart disease,n (%)** |  |  |  | 0.813 |
| **Yes** | 470 ( 5.0) | 86 (4.9) | 384 (5.0) |  |
| **No** | 8946 (95.0) | 1676 (95.1) | 7270 (95) |  |
| **stroke, n (%)** |  |  |  | 0.518 |
| **Yes** | 241 ( 2.6) | 49 (2.8) | 192 (2.5) |  |
| **No** | 9199 (97.4) | 1719 (97.2) | 7480 (97.5) |  |
| **Tinnitus, n (%)** |  |  |  | < 0.001 |
| **Yes** | 1367 (14.5) | 198 (11.2) | 1169 (15.2) |  |
| **No** | 8072 (85.5) | 1569 (88.8) | 6503 (84.8) |  |
| **Ear infections, n (%)** |  |  |  | < 0.001 |
| **Yes** | 2141 (23.4) | 327 (19.1) | 1814 (24.4) |  |
| **No** | 6996 (76.6) | 1389 (80.9) | 5607 (75.6) |  |
| **Loud noise exposure in**  **past 24-hour, n (%)** |  |  |  | 0.207 |
| **Yes** | 1196 (14.5) | 76 (12.7) | 1120 (14.6) |  |
| **No** | 7076 (85.5) | 522 (87.3) | 6554 (85.4) |  |
| **Dietary supplements , n (%)** |  |  |  | < 0.001 |
| **Yes** | 4424 (46.9) | 753 (42.6) | 3671 (47.8) |  |
| **No** | 5016 (53.1) | 1014 (57.4) | 4002 (52.2) |  |
| **Dietary calorie intake,Mean (SD),kcal/d** | 2093.6 ± 856.7 | 2031.8 ± 947.6 | 2098.3 ± 849.3 | 0.070 |
| **Dietary protein intake,Mean (SD),g/d** | 82.5 ± 36.5 | 80.3 ± 37.9 | 82.7 ± 36.4 | 0.123 |
| **Dietary carbohydrate intake ,Mean (SD),g/d** | 252.9 ± 109.3 | 251.3 ± 119.7 | 253.0 ± 108.4 | 0.708 |
| **Dietary total fat intake, Mean (SD), g/d** | 79.6 ± 40.1 | 75.1 ± 43.7 | 79.9 ± 39.8 | 0.006 |
