# Supplementary material for: L-shaped relationship between dietary niacin intake and hearing loss in United States adults: National health and nutrition examination survey
Source: PLoS One. 2025 Feb 25;20(2):e0319386. doi: 10.1371/journal.pone.0319386 (PMC11856504; doi:10.1371/journal.pone.0319386)
Supplement: S2 Table — (DOC) [file pone.0319386.s002.doc]

**S2 Table Association of dietary niacin intake with hearing loss**

| **Dietary niacin intake ,mg/day** | **NO.** | **crude** | **P_value** | **Model 1** | **P_value** | **Model 2** | **P_value** | **Model 3** | **P_value** |
| --- | --- | --- | --- | --- | --- | --- | --- | --- | --- |
| **Low-frequency hearing loss** | | | | | | | | | |
| **Quartiles** |  |  |  |  |  |  |  |  |  |
| **Q1（≤14.06）** | 1128 | 1(Ref) |  | 1(Ref) |  | 1(Ref) |  | 1(Ref) |  |
| **Q2（14.07-18.03）** | 1128 | 0.75 (0.58~0.97) | 0.026 | 0.77 (0.59~1.00) | 0.05 | 0.75 (0.57~0.98) | 0.036 | 0.77 (0.58~1.04) | 0.089 |
| **Q3（18.04-21.88）** | 1128 | 0.64 (0.5~0.83) | 0.001 | 0.67 (0.51~0.88) | 0.004 | 0.64 (0.49~0.85) | 0.002 | 0.65 (0.46~0.92) | 0.015 |
| **Q4（＞21.89）** | 1128 | 0.62 (0.47~0.8) | <0.001 | 0.62 (0.47~0.82) | 0.001 | 0.57 (0.42~0.75) | <0.001 | 0.58 (0.39~0.86) | 0.006 |
| **Speech-frequency** **hearing loss** | | | | | | | | | |
| **Quartiles** |  |  |  |  |  |  |  |  |  |
| **Q1（≤14.06）** | 1128 | 1(Ref) |  | 1(Ref) |  | 1(Ref) |  | 1(Ref) |  |
| **Q2（14.07-18.03）** | 1128 | 0.81 (0.65~1.01) | 0.067 | 0.8 (0.63~1.02) | 0.069 | 0.79 (0.62~1.01) | 0.064 | 0.84 (0.64~1.10) | 0.214 |
| **Q3（18.04-21.88）** | 1128 | 0.69 (0.55~0.86) | 0.001 | 0.65 (0.51~0.84) | 0.001 | 0.64 (0.49~0.83) | 0.001 | 0.69 (0.50~0.94) | 0.019 |
| **Q4（＞21.89）** | 1128 | 0.75 (0.60~0.93) | 0.01 | 0.67 (0.52~0.86) | 0.001 | 0.62 (0.48~0.81) | <0.001 | 0.67 (0.47~0.94) | 0.023 |
| **High-frequency hearing loss** | | | | | | | | | |
| **Quartiles** |  |  |  |  |  |  |  |  |  |
| **Q1（≤14.06）** | 1128 | 1(Ref) |  | 1(Ref) |  | 1(Ref) |  | 1(Ref) |  |
| **Q2（14.07-18.03）** | 1128 | 0.82 (0.69~0.98) | 0.025 | 0.76 (0.61~0.94) | 0.01 | 0.76 (0.61~0.94) | 0.012 | 0.75 (0.59~0.96) | 0.019 |
| **Q3（18.04-21.88）** | 1128 | 0.70 (0.59~0.83) | <0.001 | 0.59 (0.48~0.73) | <0.001 | 0.59 (0.48~0.74) | <0.001 | 0.62 (0.47~0.81) | <0.001 |
| **Q4（＞21.89）** | 1128 | 0.88 (0.74~1.04) | 0.132 | 0.77 (0.62~0.95) | 0.017 | 0.76 (0.61~0.95) | 0.017 | 0.75 (0.56~1.02) | 0.068 |

Model 1 adjusted for age,sex.

Model 2 adjusted for age,sex,tinnitus,ear infections,hypertension,diabetes,stroke,coronary heart disease.

Model 3 adjusted for age,sex,tinnitus,ear infections, hypertension,diabetes,stroke,coronary heart disease,race/ethnicity,education level,household income,marital status,body mass index,smoking status,drink status, noise exposure,hearing protection,dietary energy intake,dietary protein intake,dietary carbohydrate intake,dietary total fat intake,dietary supplements.
