# Supplementary material for: L-shaped relationship between dietary niacin intake and hearing loss in United States adults: National health and nutrition examination survey
Source: PLoS One. 2025 Feb 25;20(2):e0319386. doi: 10.1371/journal.pone.0319386 (PMC11856504; doi:10.1371/journal.pone.0319386)
Supplement: S3 Table — Model 1 adjusted for age, sex. Model 2 adjusted for age, sex, tinnitus, ear infections, hypertension, diabetes, stroke, coronary heart disease. Model 3 adjusted for age, sex, tinnitus, ear infections, hypertension, diabetes, stroke, coronary heart disease, race/ethnicity, education level, household income, marital status, body mass index, smoking status, drink status, noise exposure, hearing protection, dietary energy intake, dietary protein intake, dietary carbohydrate intake, dietary total fat intake, dietary supplements. (DOC) [file pone.0319386.s003.doc]

**S3 Table Association of dietary niacin intake with hearing loss**

| **Dietary niacin intake ,mg/day** | **NO.** | **crude** | **P_value** | **Model 1** | **P_value** | **Model 2** | **P_value** | **Model 3** | **P_value** |
| --- | --- | --- | --- | --- | --- | --- | --- | --- | --- |
| **Low-frequency hearing loss** | | | | | | | | | |
| **Quartiles** |  |  |  |  |  |  |  |  |  |
| **Q1（≤16.96）** | 1890 | 1(Ref) |  | 1(Ref) |  | 1(Ref) |  | 1(Ref) |  |
| **Q2（16.97-23.40）** | 1918 | 0.68 (0.56~0.84) | <0.001 | 0.71 (0.57~0.89) | 0.002 | 0.68 (0.55~0.85) | 0.001 | 0.73 (0.58~0.93) | 0.011 |
| **Q3（23.41-31.57）** | 1918 | 0.76 (0.62~0.93) | 0.008 | 0.79 (0.64~0.99) | 0.036 | 0.77 (0.61~0.96) | 0.019 | 0.85 (0.65~1.12) | 0.238 |
| **Q4（＞31.58）** | 1871 | 0.61 (0.49~0.75) | <0.001 | 0.75 (0.59~0.95) | 0.017 | 0.74 (0.58~0.94) | 0.015 | 0.82 (0.57~1.19) | 0.299 |
| **Speech-frequency hearing loss** | | | | | | | | | |
| **Quartiles** |  |  |  |  |  |  |  |  |  |
| **Q1（≤16.96）** | 1890 | 1(Ref) |  | 1(Ref) |  | 1(Ref) |  | 1(Ref) |  |
| **Q2（16.97-23.40）** | 1918 | 0.76 (0.63~0.90) | 0.002 | 0.74 (0.61~0.9) | 0.002 | 0.71 (0.58~0.87) | 0.001 | 0.76 (0.61~0.95) | 0.015 |
| **Q3（23.41-31.57）** | 1918 | 0.85 (0.72~1.01) | 0.063 | 0.78 (0.64~0.95) | 0.014 | 0.76 (0.62~0.93) | 0.008 | 0.83 (0.65~1.05) | 0.125 |
| **Q4（＞31.58）** | 1871 | 0.76 (0.64~0.91) | 0.002 | 0.81 (0.66~1.00) | 0.046 | 0.81 (0.66~1.01) | 0.058 | 0.9 (0.66~1.24) | 0.529 |
| **High-frequenc hearing loss** | | | | | | | | | |
| **Quartiles** |  |  |  |  |  |  |  |  |  |
| **Q1（≤16.96）** | 1890 | 1(Ref) |  | 1(Ref) |  | 1(Ref) |  | 1(Ref) |  |
| **Q2（16.97-23.40）** | 1918 | 0.82 (0.72~0.93) | 0.003 | 0.76 (0.64~0.9) | 0.001 | 0.75 (0.64~0.89) | 0.001 | 0.81 (0.67~0.97) | 0.024 |
| **Q3（23.41-31.57）** | 1918 | 1.00 (0.88~1.140) | 0.997 | 0.90 (0.76~1.07) | 0.226 | 0.90 (0.76~1.07) | 0.245 | 0.95 (0.78~1.17) | 0.652 |
| **Q4（＞31.58）** | 1871 | 0.85 (0.74~0.97) | 0.014 | 0.84 (0.7~1.01) | 0.059 | 0.85 (0.71~1.02) | 0.087 | 0.90(0.69~1.16) | 0.410 |

Model 1 adjusted for age, sex.

Model 2 adjusted for age,sex,tinnitus,ear infections,hypertension,diabetes,stroke,coronary heart disease.

Model 3 adjusted for age,sex,tinnitus,ear infections,hypertension,diabetes,stroke,coronary heart disease, race/ethnicity,education level, household income,marital status,body mass index,smoking status ,drink status, noise exposure, hearing protection,dietary energy intake,dietary protein intake,dietary carbohydrate intake,dietary total fat intake,dietary supplements.
