# Supplementary material for: L-shaped relationship between dietary niacin intake and hearing loss in United States adults: National health and nutrition examination survey
Source: PLoS One. 2025 Feb 25;20(2):e0319386. doi: 10.1371/journal.pone.0319386 (PMC11856504; doi:10.1371/journal.pone.0319386)
Supplement: Attachment — Submitted filename: Response_to_Decision_Letter_auresp_2.docx [file pone.0319386.s006.docx]

Re:  PONE-D-24-05737

**Dear Reviewers,**

We sincerely appreciate your thorough review and insightful comments on our manuscript, titled “*L-shaped Relationship Between Dietary Niacin Intake and Hearing Loss in US Adults: National Health and Nutrition Examination Survey*” (ID: PONE-D-24-05737). In response to your feedback, we have made substantial revisions to strengthen the manuscript and are pleased to resubmit it for your consideration in PLOS ONE.

Enclosed, please find a detailed document addressing each of your comments. The revised manuscript includes the necessary changes, highlighted in red for your convenience. These revisions do not affect the statistical analyses or results.To address your concerns regarding grammatical errors and formatting, we engaged a professional English editing service staffed by native English speakers. They revised the manuscript to enhance its language, fluency, and clarity. Additionally, we thoroughly reviewed the revised text to ensure that all corrections were accurate and that the core research content remained intact.

We are grateful for your time and effort in reviewing our work. Should you require further clarification or additional information, we would be happy to provide it. We sincerely hope that our study will contribute meaningfully to the academic community.

We confirm that there are no conflicts of interest related to this work and that all authors have reviewed and approved the revised manuscript for submission.

Thank you once again for your valuable feedback

Best regards,

Yours sincerely,

Xue zhang

Email: [zhangxue_jyfy@163.com](mailto:zhangxue_jyfy@163.com)

Mailing address: Affiliated Hospital of Jining Medical University, 89 Guhuai Road, Rencheng District, Jining, Shandong Province, China;

**Reviewer: 1**

Dear Prof. Felix Gumaayiri Aabebe

We deeply appreciate your thoughtful and thorough review of our manuscript. Your acknowledgment that all concerns raised during the initial review have been addressed and that there are no issues regarding dual publication, research ethics, or publication ethics means a great deal to us. Your constructive feedback has been instrumental in enhancing the quality and rigor of our work, and we are truly grateful for your time, expertise, and support throughout this process.

**Reviewer: 2**

**Dear Reviewer,**

We sincerely thank you for your detailed and insightful feedback on our manuscript. We deeply appreciate the time and effort you invested in providing such constructive comments. Your suggestions are invaluable, and we have addressed each of them comprehensively to enhance the manuscript's clarity, rigor, and accuracy. We hope the revised version meets your expectations and is suitable for publication.

**COMMENTS FOR THE AUTHOR:**

1.There are multiple grammatical errors throughout the manuscript (spacing issues, punctuation, etc) that need to be corrected.
Response 1）

We sincerely thank you for your constructive feedback regarding the grammatical errors and formatting issues in our manuscript. In response, we engaged a professional English editing service staffed by native speakers to revise the manuscript's language, fluency, and grammar, ensuring its accuracy and clarity. We carefully reviewed the revised text to confirm that no errors remain and that the core research content was preserved. We greatly appreciate your attention to detail and are confident that these revisions have strengthened the manuscript. If any further aspects require clarification or improvement, please do not hesitate to inform us. Thank you again for your valuable input.

2.Line 67 does not make sense ("has been evidence to influence...").

Response 2）

Thank you for your thorough and detailed review, as well as your valuable suggestions. We have revised this section to enhance clarity and coherence, ensuring the language accurately reflects the intended meaning and aligns with the supporting evidence. We appreciate your careful attention to this matter. The revision has been incorporated into the manuscript and highlighted in red for your convenience (see line 52, page 3). We look forward to your favorable consideration.

Modifying dietary intake has been shown to affect the risk of hearing impairment.

3.Line 78 (hypothesis rather than assumption and need to fix the tense "we hypothesize that niacin would..."). This is the same issue with tense in line 79.

Response 3）

Thank you for your valuable feedback regarding the phrasing and tense in lines 78 and 79. We have revised these sentences to improve clarity and ensure alignment with scientific conventions. We appreciate your detailed input, which has contributed significantly to enhancing the quality of our manuscript. The revisions have been incorporated into the manuscript and highlighted in red for your convenience (see lines 63–64 on page 4). We look forward to your favorable consideration.

We hypothesize that niacin intake is lower in individuals with HL. Additionally, the dose-response relationship between dietary niacin intake and hearing loss was assessed.

4.Line 91: please indicate if this was two cycles 2011-2012 and 2015-2016. It is difficult to determine if you are referring to data between 2011-2016. NHANES identifies this information as "cycles" and it should be referred to as such.
Response 4）

Thank you for your helpful comment regarding the clarification of the NHANES data cycles. We recognize the importance of using precise terminology and have revised the sentence to refer to the data as "cycles" in accordance with NHANES conventions. This revision ensures clarity about the specific NHANES cycles included in the study.

We appreciate your careful review and constructive suggestion. The revision has been incorporated into the manuscript and highlighted in red for your convenience (see lines 75–77 on page 5). We look forward to your favorable consideration.

NHANES data from the 2011–2012 and 2015–2016 cycles were used in the present cross-sectional study. Only hearing test data for adults aged 20–69 years were available for these two cycles

5.Line 146: appears information about how preexisting conditions were categorized is missing and needs to be updated.

Response 5）

Thank you for your insightful comment regarding the categorization of preexisting conditions. In response, we have revised the manuscript to provide greater clarity on how comorbidities were categorized. Specifically, we clarified that "comorbidities" include tinnitus, ear infection, hypertension, diabetes, stroke, and coronary heart disease. This revision ensures that the specific comorbidities accounted for in the analysis are clearly identified.We appreciate your careful review and constructive suggestion, which has enhanced the clarity of the manuscript. The revision has been incorporated into the manuscript and highlighted in red for your convenience (see lines 141–142 on pages 7–8). We look forward to your favorable consideration.

Model 2 was adjusted for sex, age, and comorbidities, including tinnitus, ear infections, hypertension, diabetes, stroke, and coronary heart disease.

6. Line 152: You have included information about total dietary calories, protein and carbohydates/fats. Were these also averaged similar to dietary niacin? This would need to be described in more detail.

Response 6）

Thank you for your comment regarding the averaging of total dietary calories, protein, carbohydrates, and fats, as well as your suggestion to further clarify this process. To ensure consistency with the assessment of dietary niacin intake, we have clarified that total dietary calories, protein, carbohydrates, and fats were averaged from two assessments, similar to dietary niacin. This revision enhances clarity and ensures a consistent methodological approach for these dietary variables.We appreciate your suggestion, which has improved the transparency of our methodology. The revision has been incorporated into the manuscript and highlighted in red for your convenience (see lines 128–131 on page 7 of the revised manuscript). We look forward to your favorable consideration.

Based on the dietary survey, 24-hour nutritional information was obtained for participants, including total dietary calories, protein, carbohydrates, and total fat. Similar to dietary niacin intake, these variables were averaged from two assessments to ensure consistency in the evaluation.

7. Also, you need to be specific about fat. Is this total fat or saturated fat?

Response 7）

Thank you for your comment regarding the clarification of fat intake. We have specified that the analysis refers to total fat intake, rather than saturated fat, and have made this distinction throughout the manuscript. The revised text now consistently refers to total fat intake. We appreciate your attention to this detail, which has enhanced the clarity of our study.

8.Also, how are these being represented. Grams or percentages of dietary intake?

Response 8）

Thank you for your insightful question regarding the representation of dietary intake. In response, we have clarified the units for the dietary variables in the manuscript. Specifically, dietary calorie intake is presented in kilocalories per day (kcal/d), while dietary protein, carbohydrate, and fat intakes are expressed in grams per day (g/d). Dietary niacin intake is presented in milligrams per day (mg/d). These units are now consistently specified throughout the manuscript, including in Table 1. We appreciate your attention to this detail, which has enhanced the clarity of our presentation.
