# Supplementary material for: L-shaped relationship between dietary niacin intake and hearing loss in United States adults: National health and nutrition examination survey
Source: PLoS One. 2025 Feb 25;20(2):e0319386. doi: 10.1371/journal.pone.0319386 (PMC11856504; doi:10.1371/journal.pone.0319386)
Supplement: Attachment — Submitted filename: Response to Decision Letter.docx [file pone.0319386.s005.docx]

Re:  PONE-D-24-05737

**Dear Dr. Aziz ur Rahman Muhammad and Reviewers,**

We, the co-authors, would like to express our sincere gratitude for your thorough review and valuable comments. We are confident that the revisions made in response to your suggestions have strengthened our manuscript, entitled "L-shaped relationship between dietary niacin intake and hearing loss in US adults: National Health and Nutrition Examination Survey" (ID: PONE-D-24-05737), which we are resubmitting for consideration in PLOS ONE. We are hopeful for a favorable review of the revised submission.

Enclosed, please find a document providing detailed responses to each comment. We have made the corresponding revisions to our manuscript without altering the statistical analyses or the results. We have highlighted the revised sections in red for ease of review. We look forward to your positive response to the revised paper submitted here.

Your time and attention to our research are greatly appreciated, and we stand ready to provide any further information or clarification that may be needed during the review process. It is our earnest hope that the publication of this study in your prestigious journal will make a significant contribution to the academic community.

We declare that there are no conflicts of interest concerning this work, and all authors have read and endorsed the revised manuscript for submission to PLOS ONE. Please feel free to reach out to us for any additional assistance.

Thank you and best regards.

Yours sincerely,

Xue zhang

Email: [zhangxue_jyfy@163.com](mailto:zhangxue_jyfy@163.com)

Mailing address: Affiliated Hospital of Jining Medical University, 89 Guhuai Road, Rencheng District, Jining, Shandong Province, China;

**Reviewer: 1**

Dear Reviewer,

Thank you very much for your time involved in reviewing the manuscript and your positive comments and valuable suggestions to improve the quality of our manuscript. We have proofread the manuscript carefully. And we hope the revised manuscript could be acceptable for you.

**COMMENTS FOR THE AUTHOR:**

This is an interesting manuscript on the “L-shaped relationship between dietary niacin intake and hearing loss in US adults: National Health and Nutrition Examination Survey." The manuscript is well-written showing empirical evidence and meeting all standards of quantitative research. However, in the result section, the author stated that after accounting for all potential confounders there was a significant negative association between dietary niacin intake and HL but failed to indicate those confounders and how they were accounted for.My recommendation

1.The author should list the potential confounders that were accounted for.

Response 1）

We would like to express our gratitude for your comprehensive and detailed review, which provided positive affirmation and high-quality guidance. We concur with your suggestions and have implemented substantial revisions to the manuscript. For your reference, we have amended this in the manuscript and highlighted it in red. Please refer to lines123-130 on page 7-8 ,lines197-199 on page 11 and lines 222-226 on page 12 of the revised manuscript. We look forward to your favourable consideration response.

In our study, we considered a comprehensive range of potential confounding variables to ensure the robustness and validity of our findings. The adjusted variables include:

Demographic Factors:age, sex. race/ethnicity,

Socioeconomic Status:education level,marital status,household income.

Health and Lifestyle Factors:body mass index,drink status,smoking status.

Dietary Factors:dietary energy intake, dietary protein intake, dietary carbohydrate intake, dietary fat intake, dietary supplements

Medical History:tinnitus, ear infection, hypertension, diabetes, stroke, coronary heart disease , noise exposure, hearing protection.

To account for these potential confounding variables, we employed multifactor logistic regression analysis. Each confounding variable was incorporated into the model as a covariate to adjust for its effect on the primary outcome. The effects of these potential confounding variables were controlled in this manner. Additionally, we conducted stratification and sensitivity analyses to further ensure the robustness of our findings.

2.The author should describe how the potential confounders were accounted for.

Response 2）

We are grateful for your valuable input. In our study, we took great care to identify and control for numerous potential confounding variables, thereby ensuring the accuracy and reliability of our results. The specific measures we employed are outlined below:

①Potential confounders were identified based on previous studies, clinical experience, p-values less than 0.05 in univariate analyses, and a change of more than 10% in the effect sizes of dietary niacin intake on hearing loss before and after adjusting for variables[1-6],including age; sex; ethnicity/race; education level; marital status; household income; body mass index (BMI); smoking status; alcohol consumption status; ear infections; tinnitus; hearing protection; noise exposure; hypertension; stroke; diabetes; coronary heart disease; use of dietary supplements; dietary calorie intake; dietary protein intake;dietary carbohydrate intake;dietary fat intake.

②Our study utilizes data from the National Health and Nutrition Examination Survey (NHANES), which provides comprehensive coverage, high-quality data on demographics and health behaviors, laboratory and physical examination data, and a nationally representative sample. This enables us to control for confounding variables with greater precision and comprehensiveness, thereby contributing to more reliable and accurate study conclusions.

③During the data analysis phase, we employed multifactor logistic regression analysis to incorporate the aforementioned confounders as control variables into the model, thereby minimizing their impact on the primary results. Additionally, we conducted subgroup analyses and multiple sensitivity analyses to further control for the effects of potential confounders, ensuring the accuracy and reliability of the results.

④Our methodology is informed by the studies of Huanxian Liu et al. (2022), Yi Zhang et al. (2023), and Yafei Mao et al. (2022), who also employed multifactor logistic regression analyses, subgroup analyses, and sensitivity analyses to control for confounding factors in similar research[6-8].

The reliability of our findings was ensured through the implementation of a rigorous study design and robust data analysis methods that effectively controlled for potential confounding factors.

References

1.Hearing loss prevalence and years lived with disability, 1990-2019: findings from the Global Burden of Disease Study 2019. Lancet. 2021;397(10278):996-1009. doi: 10.1016/s0140-6736(21)00516-x. PubMed PMID: 33714390; PubMed Central PMCID: PMCPMC7960691.

2.Wei X. Dietary magnesium and calcium intake is associated with lower risk of hearing loss in older adults: A cross-sectional study of NHANES. Front Nutr. 2023;10:1101764. Epub 20230314. doi: 10.3389/fnut.2023.1101764. PubMed PMID: 36998904; PubMed Central PMCID: PMCPMC10043168.

3.Agoritsas T, Merglen A, Shah ND, O'Donnell M, Guyatt GH. Adjusted Analyses in Studies Addressing Therapy and Harm: Users' Guides to the Medical Literature. Jama. 2017;317(7):748-59. doi: 10.1001/jama.2016.20029. PubMed PMID: 28241362.

4.Kernan WN, Viscoli CM, Brass LM, Broderick JP, Brott T, Feldmann E, et al. Phenylpropanolamine and the risk of hemorrhagic stroke. N Engl J Med. 2000;343(25):1826-32. doi: 10.1056/nejm200012213432501. PubMed PMID: 11117973.

5.Jaddoe VW, de Jonge LL, Hofman A, Franco OH, Steegers EA, Gaillard R. First trimester fetal growth restriction and cardiovascular risk factors in school age children: population based cohort study. Bmj. 2014;348:g14. Epub 20140123. doi: 10.1136/bmj.g14. PubMed PMID: 24458585; PubMed Central PMCID: PMCPMC3901421.

6.Liu H, Wang L, Chen C, Dong Z, Yu S. Association between Dietary Niacin Intake and Migraine among American Adults: National Health and Nutrition Examination Survey. Nutrients. 2022 Jul 25;14(15):3052. doi: 10.3390/nu14153052. PMID: 35893904; PMCID: PMC9330821.

7.Zhang Y, Lu J, Huang S, Chen Y, Fang Q, Cao Y. Sex differences in the association between serum α-Klotho and depression in middle-aged and elderly individuals: A cross-sectional study from NHANES 2007-2016. J Affect Disord. 2023 Sep 15;337:186-194. doi: 10.1016/j.jad.2023.05.073. Epub 2023 May 24. PMID: 37236270.

8.Mao Y, Li X, Zhu S, Geng Y. Association Between Dietary Fiber Intake and Risk of Depression in Patients With or Without Type 2 Diabetes. Front Neurosci. 2022 Jul 12;16:920845. doi: 10.3389/fnins.2022.920845. PMID: 36389250; PMCID: PMC9642095.

Methods:

Potential confounders were identified based on previous studies, clinical experience, p-values less than 0.05 in univariate analyses, and a change of more than 10% in the effect sizes of dietary niacin intake on hearing loss before and after adjusting for variables[5, 25-29],including age; sex; ethnicity/race; education level; marital status; household income; body mass index (BMI); smoking status; alcohol consumption status; ear infections; tinnitus; hearing protection; noise exposure; hypertension; stroke; diabetes; coronary heart disease; use of dietary supplements; dietary calorie intake; dietary protein intake;dietary carbohydrate intake;dietary fat intake;

Results :

The model was analyzed using multifactorial logistic regression, with all confounders accounted for in Model 3. The results indicated that dietary niacin intake was negatively associated with the risk of developing hearing loss.

Discussion:

The findings of this study indicated a negative correlation between dietary niacin intake and hearing loss. To ensure the reliability of the results, potential confounding variables were controlled for in Model 3 of our analyses. Additionally, the reliability and stability of the results were evaluated through multifactorial logistic regression analyses, stratification analyses, and sensitivity analyses.

**Reviewer: 2**

Dear Reviewer,

Thank you very much for your time involved in reviewing the manuscript and your positive comments and valuable suggestions to improve the quality of our manuscript. We have proofread the manuscript carefully, and rephrased the paragraphs and incorrect sentences. And we hope the revised manuscript could be acceptable for you.

**COMMENTS FOR THE AUTHOR:**

 Method Section:

1.It is still unclear how dietary niacin was obtained. Could you please provide more details?

Response 1）

We appreciate your valuable suggestions, which have enhanced the rigor of our research. Following your guidance, we have thoroughly revised the manuscript. For your reference, we have included detailed information on page 6-7, line106-119, highlighted in red for your review.

The data on dietary niacin intake were collected through 24-hour dietary recall interviews.NHANES dietary surveys were conducted by professionally trained personnel and were based on the Dietary Recall Interview measureme（https//www.cdc.gov/nchs/nhanes/guidemeasuring_guides_dri/measuringguides.htm).by converting to United States Department of Agriculture (USDA) standardized reference codes, the food intake aligned with the USDA Food and Nutrient Database for Dietary Studies (FNDDS) [22, 23]. The nutritional value of each individual's dietary intake was accurately calculated using the Computer-Assisted Dietary Interview (CADI) system and the Automated Multiple Pass Method (AMPM). The dietary information provided by the participants was assessed on two occasions by professionals in accordance with the Dietary Recall Interview Measurement Guide. The initial assessment was conducted in person at a mobile screening center, while the second assessment was conducted via telephone between three and ten days later. This approach to dietary assessment was extensively discussed at a workshop on NHANES data collection procedures and subsequently endorsed by experts in the field[24].

2.What is the goal of assessing meal frequency (line 113)? Additionally, the term "four group" in line 114 could be changed to "categorized into quartiles" for better clarity.

Response 2）

We would like to express our gratitude for your comprehensive and meticulous examination of our manuscript and the invaluable insights you have provided. We are grateful for the opportunity to elucidate and refine the manuscript in accordance with your recommendations. The objective of our study was to evaluate the relationship between dietary niacin intake and hearing loss (HL).Dietary niacin intake was assessed using a 24-hour dietary recall interview, initially conducted at a mobile examination center (MEC) and subsequently via telephone between three and ten days later. This method allowed for the evaluation of dietary intake data from two nonconsecutive days, leading to more accurate estimates of nutrient intake. Consequently, the dietary niacin intake data used in our study represented the mean of these two assessments.The term "meal frequency" was replaced with "dietary niacin intake" to more accurately reflect the objective of the study and to enhance the scientific rigour and clarity of the study. It is our hope that this change will be viewed favourably.Additionally, we greatly appreciate your valuable suggestion to change "the term 'four group' in line 114" to "categorized into quartiles" for better clarity. For a comprehensive overview, please refer to page 7, line 119-121. Thank you once again for your review and recommendations.

In this study, dietary niacin intake was averaged from two assessments. Participants were then categorized into quartiles based on their dietary niacin intake.

3.Background Section:

The explanation of how niacin will be assessed among other vitamins/micronutrients is not yet clear. Please elaborate on the importance of niacin and why it is a focus of this study.

Response 3）

We are grateful for your comprehensive review of the manuscript and the constructive feedback you have provided. We have meticulously revised the manuscript in accordance with your suggestions and have highlighted them in red. For your reference, please direct your attention to page 3, line 25-32 of the manuscript for detailed information. We eagerly anticipate your favorable response.

Hearing loss poses a significant threat to human health, with its prevalence increasing annually. Niacin (vitamin B3) is an essential B vitamin that plays a crucial role in energy metabolism and cellular repair in the body. Additionally, it exerts a protective influence on the cells of the inner ear. A correlation between dietary niacin and hearing loss has been reported; however, the results remain controversial, necessitating further investigation. This study aimed to examine the potential association between dietary niacin intake and hearing loss in U.S. adults, providing a reference for dietary preventive management of hearing loss.

4. Method Section - Noise Exposure and Hearing Protection:

Could you provide more details on how noise exposure and hearing protection were determined?

Response 4）

We are grateful for your insightful suggestions and concur with your observations. We have undertaken a thorough review of the manuscript and made the requisite changes, which we have highlighted in red. Please refer to page 8, line 138-140for further details. We reiterate our appreciation for your invaluable guidance and advice.

Noise exposure is defined as exposure to loud noise within the past 24 hours.Hearing protection is classified according to the frequency of its use during noise exposure. The categories are: always, about half the time, seldom, and never.

5.Method and Discussion Sections - Types of Hearing Loss:

Could you briefly explain the differences between low-frequency hearing loss (LFHL), speech-frequency hearing loss (SFHL), and high-frequency hearing loss (HFHL) in both the method and discussion sections?

Response 5）

We are grateful for your perceptive recommendations and have incorporated the relevant information into the Methods and Discussion section as you suggested. We look forward to your feedback and anticipate a favorable response. We have implemented this change in the manuscript and highlighted it in red. Please refer to lines 95-104 on page 6 and lines 243-248 on page 13 of the revised manuscript.

**Methods**

# In the current investigation, the outcome variables encompassed LFHL, speech-frequency hearing loss(SFHL), and HFHL. LFHL may cause difficulty in hearing low-pitched speech and environmental sounds. LFHL was delineated as the mean pure-tone audiometry (PTA) exceeding 25 dB HL at 500, 1,000, and 2,000 Hz in both ears, while SFHL may lead to difficulty in hearing and understanding everyday conversations for those with hearing impairments.SFHL was characterized by a PTA exceeding 25 dB HL at 500, 1,000, 2,000, and 4,000 Hz in both ears.HFHL may result in difficulty hearing high-pitched sounds, which can impair speech intelligibility. HFHL was defined as a PTA exceeding 25 dB HL at 3,000, 4,000, 6,000, and 8,000 Hz in both ears[18-21].

# Discussion

The impact of different frequencies of hearing loss on individuals is well documented.LFHLprimarily affects the perception of low-pitched sounds and is usually associated with middle ear problems. In contrast, SFHL affects sound perception in the speech frequency range, resulting in direct impairment of daily communication and speech comprehension. Finally, HFHL is typically caused by age-related hearing loss or noise exposure and affects the perception of high-pitched sounds[19-21, 31].

6.Logistic Regression and Covariates:

How were the covariates included in the logistic regression determined? Was it based on a p-value threshold (e.g., p<0.2) or another justification?

Response 6）

We are grateful for your comprehensive assessment of the manuscript and for the opportunity to clarify the rationale behind the inclusion of specific covariates in the logistic regression model.

1. The inclusion of covariates is based on the findings of previous literature[1-4].
2. The incorporation of covariates is based on clinical expertise.

③In the univariate regression analysis, variables with p-values less than 0.05 were included as covariates.

④Variables were identified as covariates if adjusting for them changed the effect size of dietary niacin intake on hearing loss by more than 10%[5,6].

Based on the considerations above, the covariates ultimately included in the logistic regression were: age; sex; ethnicity/race; education level; marital status; household income; body mass index (BMI); smoking status; alcohol consumption status; ear infections; tinnitus; hearing protection; noise exposure; hypertension; stroke; diabetes; coronary heart disease; use of dietary supplements; dietary calorie intake; dietary protein intake;dietary carbohydrate intake;dietary fat intake.

We referred to previous literature[7].The covariates adjusted for in the multivariable logistic regression analysis were as follows:

Model 1 adjusts age, sex;

Model 2 adjusted for sex, age and comorbidities (tinnitus, ear infection, hypertension, diabetes, stroke, and coronary heart disease)

Model 3 adjusted for all covariates（age, sex,tinnitus, ear infection, hypertension, diabetes, stroke, coronary heart disease,race/ethnicity, education level, household income,marital status,body mass index,smoking status ,drink status, noise exposure, hearing protection, use of dietary supplements, dietary calorie intake, dietary protein intake,dietary carbohydrate intake,dietary fat intake）

References

1.Liu H, Wang L, Chen C, Dong Z, Yu S. Association between Dietary Niacin Intake and Migraine among American Adults: National Health and Nutrition Examination Survey. Nutrients. 2022 Jul 25;14(15):3052. doi: 10.3390/nu14153052. PMID: 35893904; PMCID: PMC9330821.

2,Wei X. Dietary magnesium and calcium intake is associated with lower risk of hearing loss in older adults: A cross-sectional study of NHANES. Front Nutr. 2023 Mar 14;10:1101764. doi: 10.3389/fnut.2023.1101764. PMID: 36998904; PMCID: PMC10043168.

4.Hearing loss prevalence and years lived with disability, 1990-2019: findings from the Global Burden of Disease Study 2019. Lancet. 2021;397(10278):996-1009. doi: 10.1016/s0140-6736(21)00516-x. PubMed PMID: 33714390; PubMed Central PMCID: PMCPMC7960691.

5.Jaddoe VW, de Jonge LL, Hofman A, Franco OH, Steegers EA, Gaillard R. First trimester fetal growth restriction and cardiovascular risk factors in school age children: population based cohort study. BMJ. 2014 Jan 23;348:g14. doi: 10.1136/bmj.g14. PMID: 24458585; PMCID: PMC3901421.

6.Kernan WN, Viscoli CM, Brass LM, Broderick JP, Brott T, Feldmann E, Morgenstern LB, Wilterdink JL, Horwitz RI. Phenylpropanolamine and the risk of hemorrhagic stroke. N Engl J Med. 2000 Dec 21;343(25):1826-32. doi: 10.1056/NEJM200012213432501. PMID: 11117973.

7.Agoritsas T, Merglen A, Shah ND, O'Donnell M, Guyatt GH. Adjusted Analyses in Studies Addressing Therapy and Harm: Users' Guides to the Medical Literature. Jama. 2017;317(7):748-59. doi: 10.1001/jama.2016.20029. PubMed PMID: 28241362.

7.Assessment of Other Vitamins:

Why were other vitamins, such as B12 and folate, not assessed in the study?

Response 7）

To guarantee the rigor and practical relevance of our study, we concentrated on the relationship between niacin (vitamin B3) and hearing loss (HL). Hearing loss represents a significant and growing public health concern, with its prevalence increasing annually. Niacin is an essential B vitamin that plays a critical role in energy metabolism and cellular repair. There is evidence that niacin has a protective effect on inner ear cells. In light of the established link between hearing loss and damage to inner ear cells, there has been a suggestion that niacin may play a role in maintaining auditory health. However, findings in this area have been inconsistent. Consequently, we conducted a further analysis of the relationship between dietary niacin intake and HL using data from the NHANES large sample dataset, with the aim of providing additional evidence for this association[1,2].

Furthermore,In a study of U.S. adults aged 20-69 years, Edmond K. Kabagambe et al. demonstrated a U-shaped relationship between vitamin B12 and folate levels and hearing loss[3]. The main findings of the study are as follows:

This finding led us to conclude that other dietary vitamins, such as B12 and folic acid, may exert a significant influence on hearing. In future studies,we intend to broaden our investigation to further assess the correlation between these vitamins and hearing loss, with the objective of attaining a more comprehensive understanding.

1. Kim TS, Chung JW. Associations of Dietary Riboflavin, Niacin, and Retinol with Age-related Hearing Loss: An Analysis of Korean National Health and Nutrition Examination Survey Data. Nutrients. 2019 Apr 21;11(4):896. doi: 10.3390/nu11040896. PMID: 31010085; PMCID: PMC6520829.

2.Ooi TC, Ishak WS, Sharif R, Shahar S, Rajab NF, Singh DKA, Mukari SZS. Multidimensional Risk Factors of Age-Related Hearing Loss Among Malaysian Community-Dwelling Older Adults. Clin Interv Aging. 2021 Dec 8;16:2033-2046. doi: 10.2147/CIA.S340432. PMID: 34949916; PMCID: PMC8688829.

3.Kabagambe EK, Lipworth L, Labadie RF, Hood LJ, Francis DO. Erythrocyte folate, serum vitamin B12, and hearing loss in the 2003-2004 National Health And Nutrition Examination Survey (NHANES). Eur J Clin Nutr. 2018 May;72(5):720-727. doi: 10.1038/s41430-018-0101-6. Epub 2018 Jan 29. PMID: 29379142; PMCID: PMC5948125.

8.Discussion Section:

Is the inflection point of 23.26 mg/day of niacin intake comparable to current dietary guidelines for the adult population? If so, please add this information to the discussion.

Response 8）

We are grateful for your comprehensive review of the manuscript and the invaluable suggestions and guidance you have provided, which have significantly enhanced the rigor and quality of our work. We concur with your observations.We have made this change in the manuscript and highlighted it in red. Please refer to line 232-241 on page12-13 of the revised manuscript.

In accordance with the Dietary Guidelines for Americans, 2020-2025, the recommended daily allowance (RDA) for niacin is 16 mg for adult males and 14 mg for adult females. The present study identified a threshold for dietary niacin intake of 23.26 mg/day, which, although higher than the recommended levels, suggests additional health benefits beyond the RDA. Nevertheless, this intake level remains below the tolerable upper intake level (UL) of 35 mg/day, indicating its safety. Consequently, our findings align with the dietary guidelines. This study offers a novel perspective on dietary niacin intake and provides further evidence for future research and public health practices. For more information on dietary niacin guidelines, please visit:https://ods.od.nih.gov/factsheets/Niacin-Consumer/

# Discussion：

The L-shaped relationship between niacin intake and hearing protection suggests that moderate consumption may be beneficial, whereas excessive intake might not offer additional advantages.This finding is generally consistent with, but higher than, the recommended daily intake levels set forth by the U.S. Dietary Guidelines 2020-2025 (16 mg/day for men and 14 mg/day for women)[30]. This suggests that exceeding these recommended daily intake levels may confer additional health benefits.Nevertheless, an intake of 23.26 mg/day remains below the upper tolerable limit (UL) of 35 mg/day for dietary niacin, indicating that this level of intake is safe. This finding supports the possibility that niacin intake can be slightly higher than the current dietary guideline recommendation. However, further studies are needed to confirm its long-term effects and safety.

9.What is the goal of determining an L-shaped association? Please explain this in the discussion section.

Response 9）

Thank you for your thorough and detailed review, as well as for your constructive suggestions. We have diligently made the necessary revisions to the manuscript and highlighted them in red. Please refer to line 232-241 on page12-13 of the revised manuscript.

The objective of our study is to examine the correlation between dietary niacin intake and hearing loss, with a particular focus on identifying a potential L-shaped relationship. The aim is to gain insight into how varying levels of niacin intake influence hearing health, thus providing a scientific foundation for formulating evidence-based dietary recommendations. By elucidating this relationship, we aim to offer novel insights for preventing and reducing the prevalence of hearing loss and to inform the development of public health policies.

# Discussion：

The L-shaped relationship between niacin intake and hearing protection suggests that moderate consumption may be beneficial, whereas excessive intake might not offer additional advantages.This finding is generally consistent with, but higher than, the recommended daily intake levels set forth by the U.S. Dietary Guidelines 2020-2025 (16 mg/day for men and 14 mg/day for women)[30]. This suggests that exceeding these recommended daily intake levels may confer additional health benefits.Nevertheless, an intake of 23.26 mg/day remains below the upper tolerable limit (UL) of 35 mg/day for dietary niacin, indicating that this level of intake is safe. This finding supports the possibility that niacin intake can be slightly higher than the current dietary guideline recommendation. However, further studies are needed to confirm its long-term effects and safety.
